# Supplementary material for: Disorder-enabled Andreev reflection of a quantum Hall edge
Source: Nat Commun. 2023 Apr 19;14:2237. doi: 10.1038/s41467-023-37794-1 (PMC10115825; doi:10.1038/s41467-023-37794-1)
Supplement: Supplementary file 1 — Supplementary Information [file 41467_2023_37794_MOESM1_ESM.pdf]

Supplementary Information for "Disorder-enabled Andreev reflection of a quantum Hall edge"

Vladislav D. Kurilovich, Zachary M. Raines, and Leonid I. Glazman
Department of Physics, Yale University, New Haven, CT 06520, USA

SUPPLEMENTARY METHODS

Supplementary Note 1: The need for fine-tuning for an Andreev reflection off a clean superconductor

In this section, we show that the Andreev reflection of a quantum Hall edge off a clean superconductor is possible only at a single, fine-tuned value of the magnetic field (and in its immediate vicinity). For simplicity, we dispense with the effects of vortices in the present discussion. The effective Hamiltonian describing the proximitized $\nu = 2$ quantum Hall edge reads:

$$H_{\rm eff} = \sum_\sigma \int dx\, \eta^\dagger_\sigma(x)\hbar v[-i\partial_x - k_\mu]\eta_\sigma(x) + \Delta_{\rm ind}\int_0^L dx\big(\eta^\dagger_\uparrow(x)\eta^\dagger_\downarrow(x) + \eta_\downarrow(x)\eta_\uparrow(x)\big). \tag{S1}$$

The first term here is the bare Hamiltonian of the edge states; $\eta_\sigma(x)$ is the annihilation operator for a chiral electron with spin $\sigma = \uparrow$ or $\downarrow$, k_μ is the Fermi wave vector of the edge states, and v is their propagation velocity at the NS interface [cf. Eq. (4) of the main text]. The second term in Eq. (S1) describes the proximity-induced pairing of electrons at the edge. We assume that pairing potential $\Delta_{\rm ind}$ is uniform across the proximitized segment of length L. This reflects the absence of disorder in the superconductor (we also assume that the interface is ideal). The estimate for $\Delta_{\rm ind}$ depends on the transparency of the interface; it interpolates between $\Delta_{\rm ind} \sim p_F\nu_{\rm M}(\partial_y\Phi)^2t^2/\hbar^2$ in the tunneling limit and $\Delta_{\rm ind} \sim \Delta$ in the limit of a highly transparent interface (the notations are similar to those in the main text). We neglect the Zeeman effect in Eq. (S1) (although all of the qualitative conclusions remain intact if one accounts for the Zeeman splitting in the calculations).

In the gauge with the vector potential vanishing at the interface ($y = 0$), the edge state position is $y_{\rm c} = l_B^2 k_\mu$, where $l_B = \sqrt{\hbar c/eB}$ is the magnetic length.

To verify that the efficient Andreev reflection requires fine-tuning of B, we find the conductance G in the geometry of Fig. 1(a) of the main text. This requires solving a scattering problem for a chiral electron impinging on the proximitized segment. At the Fermi level, the Schrödinger equation reads $i\hbar v\partial_x\psi(x) = -\hbar v k_\mu\tau_z\psi(x) + \Delta_{\rm ind}\tau_x\psi(x)$. Here, $\psi(x) = (a_{\rm e}(x), a_{\rm h}(x))^T$ is a two-component Nambu spinor, and $\tau_{x,z}$ are the Pauli matrices acting in the Nambu space. The initial condition is $\psi(0) = (1,0)^T$. The conductance is related to the components of the wave function at $x = L$, $G = G_Q(|a_{\rm e}(L)|^2 - |a_{\rm h}(L)|^2)$. Upon solving the Schrödinger equation, we obtain:

$$G = G_Q\Big[1 - \frac{2(\Delta_{\rm ind}/\hbar v)^2}{k_\mu^2 + (\Delta_{\rm ind}/\hbar v)^2}\sin^2\Big(L\sqrt{(\Delta_{\rm ind}/\hbar v)^2 + k_\mu^2}\Big)\Big]. \tag{S2}$$

We note that G oscillates with L. The oscillations stem from the particle-hole conversion. The amplitude of oscillations is determined by the comparison between k_μ and $\Delta_{\rm ind}/\hbar v$. It reaches the maximum value of G_Q under the condition $k_\mu = 0$. The latter is realized when $y_{\rm c} = 0$. The conclusion that the Andreev reflection requires $y_{\rm c} = 0$ agrees with the qualitative discussion presented in the main text.

The condition $k_\mu(B) = 0$ is satisfied at a single value B_0 of the magnetic field. Detuning of B from B_0 results in $k_\mu(B) \neq 0$ and thus leads to the suppression of the Andreev reflection. We estimate the variations of k_μ with B for the simplest case of strong (but smooth on the scale l_B) inhomogeneity of the density of 2DEG. In that case, the thermodynamic density of states (averaged over the area of 2DEG) is independent of the magnetic field, and therefore the Fermi level does not vary with B. Then, deviation of k_μ from zero with B can be estimated as $k_\mu(B) \sim l_B^{-1}(B - B_0)/B_0$. On the other hand, an appreciable Andreev reflection occurs only if $|k_\mu(B)| \lesssim \Delta_{\rm ind}/\hbar v$, see Eq. (S2). Combining the latter condition with our estimate for k_μ, we find that the Andreev reflection is effective in a narrow interval of fields, $|B - B_0|/B_0 \lesssim \Delta_{\rm ind}/\hbar\omega_{\rm c}$; here we used the relation[1] $v \sim l_B\omega_{\rm c}$ applicable in the case of a low filling factor, $\nu = 2$. The ratio $\Delta_{\rm ind}/\hbar\omega_{\rm c}$ is typically small even if one estimates $\Delta_{\rm ind} \sim \Delta$. For example, $\Delta \sim 10\,{\rm K}$ and $\hbar\omega_{\rm c} \sim 100\,{\rm K}$ in the experiment [S3]; we then find $|B - B_0|/B_0 \lesssim 0.1$. This confirms that the fine-tuning of magnetic field is required for an appreciable particle-hole conversion in the case of a clean superconductor.

Away from the found ("fine-tuned") field range, the probability $p_{\rm AR}$ of the Andreev reflection is small. One can estimate it as $p_{\rm AR} \sim (\Delta_{\rm ind}/\hbar\omega_{\rm c})^2$ for a generic position of the chemical potential in the gap between the Landau levels. For the experimental parameters $\Delta \sim 10\,{\rm K}$ and $\hbar\omega_{\rm c} \sim 100\,{\rm K}$ [S3], we obtain $p_{\rm AR} \sim 0.01 \ll 1$. This illustrates the suppression of the Andreev reflection in the absence of disorder.

---

[1] The edge state velocity at the NS interface depends on a number of microscopic parameters, and its evaluation is beyond the scope of our work. In our estimates, we assume that v is comparable to edge state velocity at the interface with an insulator. This is supported by a numerical evaluation of v performed in Ref. S3 for the experimentally relevant case.

Supplementary Note 2: Derivation of $\langle\Theta^2\rangle$ for the forward scattering phase Θ

Here we present the derivation of $\langle\Theta^2\rangle$ for the forward scattering phase Θ accumulated by an electron across a short proximitized segment. We can obtain an expression for Θ similarly to how we found the amplitude $A_{\rm h}$, see Eq. (7) of the main text. By treating $H_{\rm prox}$ in Eq. (3) as a perturbation, we find:

$$\Theta = \frac{(\partial_y \Phi)^2 t^2}{\hbar v}\int_0^L dx_1 dx_2 e^{ik_\mu(x_1-x_2)}\partial^2_{y_1 y_2}\mathcal{G}_{\rm ee}(x_1,x_2). \tag{S3}$$

Here $\mathcal{G}_{\rm ee}(x_1,x_2)=\mathcal{G}_{\rm ee}(\boldsymbol{r}_1,\boldsymbol{r}_2|E=0)|_{y_{1,2},z_{1,2}=0}$ is the normal component of the superconductor Green's function. It is easy to verify using Eq. (22) of the main text that $\langle\Theta\rangle=0$. An expression for $\langle\Theta^2\rangle$ can be obtained similarly to how we found $\langle|A_{\rm h}|^2\rangle$. A counterpart of Eq. (30) is

$$\begin{aligned}\langle\Theta^2\rangle =& \frac{2\pi\nu_{\rm M}(\partial_y\Phi)^4 t^4 p_F^2}{\hbar^5 v^2}\int_0^L dx_1 dx_2 dx_3 dx_4 e^{ik_\mu(x_1-x_2)}e^{-ik_\mu(x_3-x_4)}\int \frac{\epsilon d\epsilon}{\Delta^2+\epsilon^2}\frac{\epsilon' d\epsilon'}{\Delta^2+\epsilon'^2}\\ &\times \mathrm{Re}\,\mathcal{D}_{\rm D}(x_1,x_2|\epsilon-\epsilon')\left[\delta(x_1-x_3)\delta(x_2-x_4)+\delta(x_1-x_4)\delta(x_2-x_3)\right],\end{aligned} \tag{S4}$$

where we also used Eq. (31) for functions V. Using Eq. (33) for $\mathcal{D}_{\rm D}(x_1,x_2|\epsilon-\epsilon')$, we can rewrite the above expression as

$$\langle\Theta^2\rangle = \frac{\pi^2\nu_{\rm M}(\partial_y\Phi)^4 t^4 p_F^2}{2\hbar^5 v^2 D}\int_0^L \frac{dx_1 dx_2}{|x_1-x_2|}e^{-|x_1-x_2|/\xi}\left[1+\cos[2k_\mu(x_1-x_2)]\right]. \tag{S5}$$

The distance between points x_1 and x_2 here does not exceed the coherence length ξ. The latter satisfies $\xi \ll l_B \lesssim |k_\mu|^{-1}$ for a type II superconductor in field $B\ll H_{\rm c2}$. These estimates mean that the argument of cosine in Eq. (S5) is small, allowing one to approximate $\cos[2k_\mu(x_1-x_2)]=1$. Then, the right hand side of Eq. (S5) becomes identical to that of Eq. (35) for $\langle|A_{\rm h}|^2\rangle$. As a result, we obtain

$$\langle\Theta^2\rangle = \frac{L}{l_{\rm A}}, \tag{S6}$$

where $l_{\rm A}$ is given by Eq. (8) of the main text.

Supplementary Note 3: Derivation of the conductance correlation function

Here we present the derivation of the conductance correlation function $\mathcal{C}(\delta n, \delta B) = \langle\langle G(n,B)\cdot G(n+\delta n, B+\delta B)\rangle\rangle$, which we use to obtain Eqs. (16) and (17) of the main text.

To start with, we briefly discuss the main mechanism leading to the loss of correlation between the values of G at parameters (n,B) and $(n+\delta n, B+\delta B)$, respectively. Firstly, the variation $(\delta n, \delta B)$ shifts the Fermi momentum of chiral electrons by $\delta k_\mu(\delta n, \delta B)$. As discussed after Eq. (15) of the main text, this affects the phases of the Andreev amplitudes $\alpha(x)$. The phases are also affected by the change in the diamagnetic current flowing along the superconductor's surface. The two effects can be accounted for by adding the phase factor to the Andreev amplitude, $\alpha(x)\to\alpha(x)e^{2i\delta k_\mu^{(\mathrm{tot})}x}$, where

$$\delta k_\mu^{(\mathrm{tot})} = \delta k_\mu - \frac{1}{2}\delta(\partial_x\varphi) \tag{S7}$$

and $\partial_x\varphi$ is the gradient of the order parameter phase associated with the diamagnetic current. The variation $(\delta n, \delta B)$ also affects the magnitudes $|\alpha(x)|$ and $|\vartheta(x)|$. The reason is the dependence of $\partial_y\Phi$ and v in Eq. (7) of the main text and Eq. (S3) on n and B. The magnitudes change as $|\alpha(x)|\to(1+\delta g/g)|\alpha(x)|$ and $|\vartheta(x)|\to(1+\delta g/g)|\vartheta(x)|$, where we used the relation for g presented after Eq. (8) in the main text.

To find $\mathcal{C}(\delta n,\delta B)$, we use Eq. (10) of the main text to compare the results of the wave function evolution across the proximitized segment at parameters (n,B) and $(n+\delta n, B+\delta B)$. We denote the components of the wave function by $a_\mathrm{e}(x)$, $a_\mathrm{h}(x)$ and $b_\mathrm{e}(x)$, $b_\mathrm{h}(x)$ for the respective sets of parameters. The corresponding evolution equations read

$$i\frac{\partial}{\partial x}\begin{pmatrix} a_\mathrm{e}(x)\\ a_\mathrm{h}(x)\end{pmatrix} = \begin{pmatrix} -\vartheta(x) & \alpha^\star(x)\\ \alpha(x) & \vartheta(x)\end{pmatrix}\begin{pmatrix} a_\mathrm{e}(x)\\ a_\mathrm{h}(x)\end{pmatrix}, \tag{S8}$$

$$i\frac{\partial}{\partial x}\begin{pmatrix} b_\mathrm{e}(x)\\ b_\mathrm{h}(x)\end{pmatrix} = \left(1+\frac{\delta g}{g}\right)\begin{pmatrix} -\vartheta(x) & \alpha^\star(x)e^{-2i\delta k_\mu^{(\mathrm{tot})}x}\\ \alpha(x)e^{2i\delta k_\mu^{(\mathrm{tot})}x} & \vartheta(x)\end{pmatrix}\begin{pmatrix} b_\mathrm{e}(x)\\ b_\mathrm{h}(x)\end{pmatrix}. \tag{S9}$$

We can represent $\mathcal{C}(\delta n,\delta B)$ in terms of the wave function components as

$$\mathcal{C}(\delta n,\delta B) = \langle\langle G^2\rangle\rangle\frac{\langle\langle |a_\mathrm{h}(L)|^2\cdot|b_\mathrm{h}(L)|^2\rangle\rangle}{\langle\langle |a_\mathrm{h}(L)|^2\cdot|a_\mathrm{h}(L)|^2\rangle\rangle}. \tag{S10}$$

To determine $\langle\langle |a_\mathrm{h}(L)|^2\cdot|b_\mathrm{h}(L)|^2\rangle\rangle$, we derive a system of differential equations describing the evolution with x of the correlators $\langle\langle a_i^\star(x)a_j(x)\cdot b_k^\star(x)b_l(x)\rangle\rangle$, where $i,j,k,l=\mathrm{e},\mathrm{h}$. In fact, a closed system of equations can be obtained using Eq. (9) of the main text and following the approach described in Ref. S1. The system has a particularly simple form in terms of the following variables:

$$c_0(x) = \langle\langle |a_\mathrm{h}(x)|^2\cdot|b_\mathrm{h}(x)|^2\rangle\rangle + e^{-2\left(1+(1+\frac{\delta g}{g})^2\right)\frac{x}{l_\mathrm{A}}}/4, \tag{S11}$$

$$c_+(x) = \mathrm{Re}\,\langle\langle a_\mathrm{e}^\star(x)a_\mathrm{h}(x)\cdot b_\mathrm{h}^\star(x)b_\mathrm{e}(x)\rangle\rangle, \tag{S12}$$

$$c_-(x) = \mathrm{Im}\,\langle\langle a_\mathrm{e}^\star(x)a_\mathrm{h}(x)\cdot b_\mathrm{h}^\star(x)b_\mathrm{e}(x)\rangle\rangle. \tag{S13}$$

We obtain

$$\frac{\partial}{\partial x}\begin{pmatrix} c_0(x)\\ c_+(x)\\ c_-(x)\end{pmatrix} = \frac{1}{l_\mathrm{A}}\begin{pmatrix} -2\big(1+\big(1+\frac{\delta g}{g}\big)^2\big) & 2\big(1+\frac{\delta g}{g}\big) & 0\\ 4\big(1+\frac{\delta g}{g}\big) & -\big(1+\big(1+\frac{\delta g}{g}\big)^2\big)-2\big(\frac{\delta g}{g}\big)^2 & 2\delta k_\mu^{(\mathrm{tot})}l_\mathrm{A}\\ 0 & -2\delta k_\mu^{(\mathrm{tot})}l_\mathrm{A} & -\big(1+\big(1+\frac{\delta g}{g}\big)^2\big)-2\big(\frac{\delta g}{g}\big)^2\end{pmatrix}\begin{pmatrix} c_0(x)\\ c_+(x)\\ c_-(x)\end{pmatrix} \tag{S14}$$

(we also made a gauge transformation $b_{\mathrm{e}/\mathrm{h}}(x)\to e^{\mp i\delta k_\mu^{(\mathrm{tot})}x}b_{\mathrm{e}/\mathrm{h}}(x)$ when deriving the system). The initial conditions are $c_0(0)=1/4$, $c_\pm(0)=0$.

Let us assume that $\delta k_\mu^{(\mathrm{tot})}l_\mathrm{A}\ll 1$ and $\delta g/g\ll 1$. Under these conditions, system (S14) can be analyzed with the help of the perturbation theory. At $\delta g=0$ and $\delta k_\mu^{(\mathrm{tot})}=0$, the 3×3 matrix on the right hand side of Eq. (S14) has an eigenvalue $\omega=0$. The zero eigenvalue corresponds to the steady state solution of the Fokker-Planck equation, see Eq. (11) of the main text. The respective eigenvector is $(1,2,0)^T$. The correction to $\omega=0$ due to finite $\delta k_\mu^{(\mathrm{tot})}$ and δg is of the second order in these parameters:

$$\omega = -\frac{4}{3}(\delta k_\mu^{(\mathrm{tot})})^2 l_\mathrm{A} - \frac{8}{3}\left(\frac{\delta g}{g}\right)^2\frac{1}{l_\mathrm{A}}. \tag{S15}$$

Using this expression, we find the solution of system (S14) at $x\gg l_\mathrm{A}$:

$$\begin{pmatrix} c_0(x)\\ c_+(x)\\ c_-(x)\end{pmatrix} = \frac{1}{12}\begin{pmatrix}1\\2\\0\end{pmatrix}\exp\Big[-\frac{4}{3}(\delta k_\mu^{(\mathrm{tot})})^2 l_\mathrm{A}x - \frac{8}{3}\Big(\frac{\delta g}{g}\Big)^2\frac{x}{l_\mathrm{A}}\Big]. \tag{S16}$$

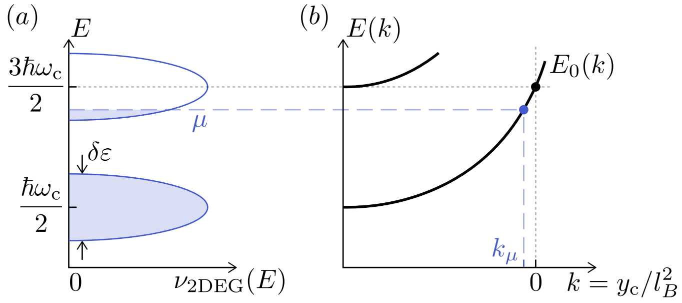

FIG. 1. **a**. Sketch of the density of states $\nu_{2DEG}(E)$ in the bulk of the 2DEG. Blue semicircles depict the Landau levels broadened by the short-ranged disorder [S5]. In our estimates, we assume that the broadening is small, $\delta\varepsilon \ll \hbar\omega_c$, and consider the case of chemical potential μ lying in the first (broadened) Landau level. The depicted configuration realizes the $\nu = 2$ quantum Hall state. **b.** Sketch of the edge state dispersion relation for the lowest Landau level, $E_0(k)$. The wave vector $k = y_c/l_B^2$ is related to the edge state position y_c. The shown dispersion relation corresponds to the hard-wall boundary condition imposed on wave function at the interface, $y = 0$. Note that $E_0(k = 0) = 3\hbar\omega_c/2$ (see the dotted lines) [S6]. The latter property justifies the expansion used to obtain Eq. (S22).

Setting $x = L$ and using Eqs. (S11) and (S10), we obtain

$$\mathcal{C}(\delta n, \delta B) = \langle\langle G^2 \rangle\rangle \exp\Big[-\frac{4}{3}(\delta k_\mu^{(\mathrm{tot})})^2 l_A L - \frac{8}{3}\left(\frac{\delta g}{g}\right)^2 \frac{L}{l_A}\Big]. \tag{S17}$$

We now apply the general result (S17) to find the conductance correlation function with density $\mathcal{C}(\delta n)$. The change of the wave vector k_μ upon the variation δn can be expressed as

$$\delta k_\mu = \frac{\partial \mu}{\partial n}\frac{\delta n}{\hbar v}, \tag{S18}$$

where $\partial\mu/\partial n$ is the inverse compressibility of the quantum Hall state. The influence of δn on g can be disregarded provided $l_A \gg l_B = \sqrt{\hbar c/eB}$. Assuming the latter condition to be satisfied, we dispense with the second term in the square brackets of Eq. (S17). Then, substituting Eq. (S18) in Eq. (S17) we arrive to Eqs. (16) and (17) of the main text.

Let us also consider the case in which B is varied, in addition to n. To find the correlation function $\mathcal{C}(\delta n, \delta B)$, we need to relate $\delta k_\mu^{(\mathrm{tot})}$ and $\delta g/g$ to the variations δn and δB [see Eq. (S17)]. The respective relations acquire a simple form in the limit of small disorder-induced broadening of the Landau levels, $\delta\varepsilon \ll \hbar\omega_c$, where ω_c is the cyclotron frequency [see Fig. 1]. We concentrate on this limit in the remainder of the section.

We start by finding $\delta k_\mu^{(\mathrm{tot})}(\delta n, \delta B)$. There are two contributions to this quantity, cf. Eq. (S7). One contribution is associated with the variation of the diamagnetic current in the superconductor; we can estimate it as $\frac{1}{2}\delta(\partial_x\varphi) \sim \frac{1}{\lambda}(\delta B/B)$, where λ is the London penetration depth [S4]. Another contribution describes the change of the Fermi momentum of chiral electrons δk_μ due to the variations δn and δB. To the linear order, we can represent δk_μ as

$$\delta k_\mu(\delta n, \delta B) = c_n \delta n + c_B \delta B. \tag{S19}$$

Here $c_n = (\partial\mu/\partial n)/\hbar v$ [cf. Eq. (S18)]. The coefficient c_B can be related to c_n. To do that, it is convenient to consider how k_μ changes with n and B along a fixed direction in the $(\delta n, \delta B)$-plane. A special direction is the one in which the Landau level filling factor $\nu(n, B) = n/n_\phi$ is preserved [here $n_\phi = B/\phi_0$ and $\phi_0 = hc/e$ is the flux quantum]. To keep the filling factor fixed, n should vary together with B as $\delta n(\delta B) = \nu(n, B)\delta B/\phi_0$. In fact, we will see shortly that, for small broadening of the Landau levels, k_μ should remain approximately *unchanged* under such a variation, $\delta k_\mu(\delta n(\delta B), \delta B) = 0$. This fixes $c_B = -\frac{\nu(n,B)}{\phi_0} c_n$ in Eq. (S19). Therefore,

$$\delta k_\mu(\delta n, \delta B) = \frac{1}{\hbar v}\frac{\partial\mu}{\partial n}\Big[\delta n - \frac{\nu(n, B)}{\phi_0}\delta B\Big]. \tag{S20}$$

This is the expression presented for $\delta k_\mu(\delta n, \delta B)$ in the main text.

To finish the derivation, we need to demonstrate that k_μ remains approximately unchanged under variations of n and B preserving the filling factor $\nu(n, B)$. To this end, in Fig. 1 we plot the spectrum of the Landau levels. The energies of the Landau levels bend upwards as the cyclotron orbits approach the interface; this describes the formation of the edge states. We fix the gauge in which the vector potential vanishes at the interface, $y = 0$. In this gauge, the edge state position is $y_c = l_B^2 k$, where k is the wave vector of the edge state and $l_B = \sqrt{\hbar c/eB}$ is the magnetic length.

In the regime of small Landau level broadening, $\delta\varepsilon \ll \hbar\omega_c$, the chemical potential μ is within one of the broadened Landau levels, see Fig. 1. For concreteness, let us assume that μ is close to $E_1 = 3\hbar\omega_c/2$ (and yet lies below this value,

as required for realization of $\nu = 2$ quantum Hall state). The specific position of μ depends on $\nu(n, B)$. We can represent this dependence as

$$\mu = \frac{3}{2}\hbar\omega_{\rm c} + \delta\varepsilon \cdot f(\nu(n, B)), \tag{S21}$$

where f is a dimensionless function of $\nu(n, B)$ (its particular form is inconsequential for the present derivation). The broadening can be estimated as $\delta\varepsilon = \beta_0\hbar\omega_{\rm c}/\sqrt{\omega_{\rm c}\tau_{\rm 2DEG}}$, where $\tau_{\rm 2DEG}$ is the mean free time of carriers in the 2DEG and β_0 is a numerical constant [S5]. Alternatively, we can relate μ to the Fermi wave vector of chiral electrons k_μ as $\mu = E_0(k_\mu)$, where $E_0(k)$ is the edge state dispersion relation. In the relevant range of k, one can expand $E_0(k_\mu) = 3\hbar\omega_{\rm c}/2 + \beta_1\hbar\omega_{\rm c} l_B k_\mu + \ldots$, where β_1 is a number ~ 1 [see Fig. 1]. Therefore, to the linear order in k_μ,

$$\mu = \frac{3}{2}\hbar\omega_{\rm c} + \beta_1\hbar\omega_{\rm c} l_B k_\mu. \tag{S22}$$

Combining the two representations for μ [Eq. (S21) and (S22)], we find

$$k_\mu = \frac{\beta_1}{\beta_0}\sqrt{\frac{m}{\hbar\tau_{\rm 2DEG}}} f(\nu(n, B)). \tag{S23}$$

To obtain this relation, we used $\omega_{\rm c} = eB/mc$. Notice that, with the considered accuracy, k_μ depends *only* on the filling factor $\nu(n, B) = \phi_0 n/B$. Therefore, $\delta k_\mu = 0$ for $\delta\nu(n, B) = 0$, as it was assumed in the derivation of Eq. (S20).

Finally, let us find the variation $\delta g/g$. Here we focus on the dependence $g(B)$ [as discussed after Eq. (S18), the variation of g with n is negligible]. An expression for $\delta g/g$ can be worked out by the power counting. To start with, we note that, up to the B-independent factors, one can represent $g \propto (\partial_y\Phi)^2/v$ [see the in-line equation after Eq. (8) of the main text]. Here $v \sim \omega_{\rm c} l_B \propto B^{1/2}$ is the edge state velocity, and $\Phi(y)$ is the transverse component of the edge state wave function. We estimate $(\partial_y\Phi)^2 \sim 1/l_B^3 \propto B^{3/2}$. Thus, $g \propto B$, which leads to $\delta g/g = \delta B/B$. This result is valid as long as $\delta\varepsilon/\hbar\omega_{\rm c} \ll 1$.

Supplementary Note 4: Derivation of Eq. (18) for $\mathcal{C}_{\mathrm{jump}}(d)$

In this section, we present details of the derivation of Eq. (18) for the variance of the conductance jumps $\mathcal{C}_{\mathrm{jump}}(d) = \langle(\delta G)^2\rangle$. We assume the proximitized segment to be long throughout the section, $L \gg l_{\mathrm{A}}$.

To find $\mathcal{C}_{\mathrm{jump}}(d)$, we compare the results of the wave function evolution across the proximitized segment before and after a vortex has entered the superconductor. We denote the wave function components as $a_{\mathrm{e}}(x)$ and $a_{\mathrm{h}}(x)$ before the vortex entrance, and as $b_{\mathrm{e}}(x)$ and $b_{\mathrm{h}}(x)$ after it. The corresponding evolution equations are given by

$$i\frac{\partial}{\partial x}\begin{pmatrix}a_{\mathrm{e}}(x)\\ a_{\mathrm{h}}(x)\end{pmatrix} = \begin{pmatrix}-\vartheta(x) & \alpha^\star(x)\\ \alpha(x) & \vartheta(x)\end{pmatrix}\begin{pmatrix}a_{\mathrm{e}}(x)\\ a_{\mathrm{h}}(x)\end{pmatrix}, \qquad i\frac{\partial}{\partial x}\begin{pmatrix}b_{\mathrm{e}}(x)\\ b_{\mathrm{h}}(x)\end{pmatrix} = \begin{pmatrix}-\vartheta(x) & \alpha^\star(x)e^{i\delta\varphi(x-x_{\mathrm{v}})}\\ \alpha(x)e^{-i\delta\varphi(x-x_{\mathrm{v}})} & \vartheta(x)\end{pmatrix}\begin{pmatrix}b_{\mathrm{e}}(x)\\ b_{\mathrm{h}}(x)\end{pmatrix}. \tag{S24}$$

Here $\delta\varphi(x-x_{\mathrm{v}}) = \pi + 2\arctan([x-x_{\mathrm{v}}]/d)$ is the phase induced by the entered vortex (we assume that pinning in the superconductor is strong enough so that the entrance of the vortex does not affect the preexisting vortex distribution). As mentioned in the main text, d is the distance between the vortex core and the interface, and x_{v} is the core's coordinate along the x-direction.

The variance of the conductance jumps can be expressed in terms of the wave functions components as:

$$\mathcal{C}_{\mathrm{jump}}(d) = 2\langle\langle G^2\rangle\rangle\left[1 - \frac{\langle\langle|a_{\mathrm{h}}(L)|^2\cdot|b_{\mathrm{h}}(L)|^2\rangle\rangle}{\langle\langle|a_{\mathrm{h}}(L)|^2\cdot|a_{\mathrm{h}}(L)|^2\rangle\rangle}\right]. \tag{S25}$$

To find $\langle\langle|a_{\mathrm{h}}(L)|^2\cdot|b_{\mathrm{h}}(L)|^2\rangle\rangle$, we derive a system of equations for correlators $\langle\langle a_i^\star(x)a_j(x)\cdot b_k^\star(x)b_l(x)\rangle\rangle$ similarly to how we did it in Sec. . The system reads

$$\frac{\partial}{\partial x}\begin{pmatrix}c_0(x)\\ c_+(x)\\ c_-(x)\end{pmatrix} = \frac{1}{l_{\mathrm{A}}}\begin{pmatrix}-4 & 2 & 0\\ 4 & -2 & -l_{\mathrm{A}}\partial_x\delta\varphi(x-x_{\mathrm{v}})\\ 0 & l_{\mathrm{A}}\partial_x\delta\varphi(x-x_{\mathrm{v}}) & -2\end{pmatrix}\begin{pmatrix}c_0(x)\\ c_+(x)\\ c_-(x)\end{pmatrix}. \tag{S26}$$

Here variable $c_0(x) = \langle\langle|a_{\mathrm{h}}(x)|^2\cdot|b_{\mathrm{h}}(x)|^2\rangle\rangle + e^{-4x/l_{\mathrm{A}}}/4$, whereas variables $c_\pm(x)$ are defined in the same way as in Eqs. (S12) and (S13).

System of equations (S26) can be solved analytically in the two limiting cases, $d \ll l_{\mathrm{A}}$ and $d \gg l_{\mathrm{A}}$. Let us start with the former case. The condition $d \ll l_{\mathrm{A}}$ means that the kink in the superconducting phase $\delta\varphi(x-x_{\mathrm{v}})$ is narrow. This suggests one to approximate

$$\partial_x\delta\varphi(x-x_{\mathrm{v}}) = 2\pi\cdot\frac{1}{\pi}\frac{d}{d^2+(x-x_{\mathrm{v}})^2} \approx 2\pi\delta(x-x_{\mathrm{v}}) \tag{S27}$$

in Eq. (S26). However, such an approximation is too crude. Indeed, it can be easily verified that the vector $(c_0(x), c_+(x), c_-(x))^T$ does not change across $x = x_{\mathrm{v}}$ if we replace $\partial_x\delta\varphi(x-x_{\mathrm{v}}) \to 2\pi\delta(x-x_{\mathrm{v}})$. Thus, $\mathcal{C}_{\mathrm{jump}}(d) = 0$ to the zeroth order in d/l_{A}.

The leading in d/l_{A} result for $\mathcal{C}_{\mathrm{jump}}(d)$ can be obtained in the following way. First of all, we go to a rotating frame in Eq. (S26):

$$\begin{pmatrix}c_0(x)\\ c_+(x)\\ c_-(x)\end{pmatrix} = \exp\left[\begin{pmatrix}0 & 0 & 0\\ 0 & 0 & -\delta\varphi(x-x_{\mathrm{v}})\\ 0 & \delta\varphi(x-x_{\mathrm{v}}) & 0\end{pmatrix}\right]\begin{pmatrix}\tilde{c}_0(x)\\ \tilde{c}_+(x)\\ \tilde{c}_-(x)\end{pmatrix} \tag{S28}$$

(we choose the frame in such a way that terms $\propto \partial_x\delta\varphi(x-x_{\mathrm{v}})$ cancel on two sides of the equation after the transformation). In this frame, the "scatterer" associated with the vortex is described by a local perturbation of magnitude $\sim 1/l_{\mathrm{A}}$ and width $\sim d$. It can be treated using an analog of Born approximation. A straightforward calculation leads to

$$\langle\langle|a_{\mathrm{h}}(L)|^2\cdot|b_{\mathrm{h}}(L)|^2\rangle\rangle = \langle\langle|a_{\mathrm{h}}(L)|^2\cdot|a_{\mathrm{h}}(L)|^2\rangle\rangle\left(1 - \frac{16\pi}{3}\frac{d}{l_{\mathrm{A}}}\right). \tag{S29}$$

Using this expression in Eq. (S25), we obtain the result presented in the first line of Eq. (18) of the main text.

Now we consider the limit of $d \gg l_{\mathrm{A}}$. In this limit, we can account for $\partial_x\delta\varphi(x-x_{\mathrm{v}})l_{\mathrm{A}}$ in system (S26) with the help of the adiabatic approximation. Using the similarity of system (S26) to system (S14) (taken at $\delta g = 0$), we find by generalizing Eq. (S16):

$$\begin{pmatrix}c_0(x)\\ c_+(x)\\ c_-(x)\end{pmatrix} \approx \frac{1}{12}\begin{pmatrix}1\\ 2\\ 0\end{pmatrix} e^{-\frac{1}{3}\int_0^x[\partial_x\delta\varphi(x'-x_{\mathrm{v}})l_{\mathrm{A}}]^2dx'}. \tag{S30}$$

Taking $x = L \gg l_{\mathrm{A}}$, computing the integral in the exponent, and using the definition of a variable $c_0(x)$, we find

$$\langle\langle|a_{\mathrm{h}}(L)|^2\cdot|b_{\mathrm{h}}(L)|^2\rangle\rangle = \langle\langle|a_{\mathrm{h}}(L)|^2\cdot|a_{\mathrm{h}}(L)|^2\rangle\rangle\exp\left[-\frac{2\pi l_{\mathrm{A}}}{d}\right] \approx \langle\langle|a_{\mathrm{h}}(L)|^2\cdot|a_{\mathrm{h}}(L)|^2\rangle\rangle\left(1 - \frac{2\pi l_{\mathrm{A}}}{d}\right). \tag{S31}$$

Substituting this expression in Eq. (S25), we obtain the result presented in the second line of Eq. (18) of the main text.

Supplementary Note 5: Estimate of coefficient b in the expression for inelastic scattering rate

In this section, we elucidate the dependence on disorder of the coefficient b in the inelastic scattering rate $\tau_{\rm in}^{-1}(E) \sim b\,E^2$. We consider an electron elevated above the Fermi level by $E_{k_1} > 0$. Here k_1 is the electron's initial momentum; E_k encodes the dispersion of chiral electrons, $E_k = \hbar v(k - k_\mu) + \hbar^2(k-k_\mu)^2/(2\tilde{m}) + \dots$, and parameter $\tilde{m}$ determines the curvature of the dispersion relation. The total scattering rate due to the inelastic pair collisions $k_1 + k_2 \to p_1 + p_2$ can be expressed as

$$\frac{1}{\tau_{\rm in}(E_{k_1})} = \frac{2\pi}{\hbar} \sum_{k_2, p_1, p_2} |M^{k_1,k_2}_{p_1,p_2}|^2 \delta(E_{k_1} + E_{k_2} - E_{p_1} - E_{p_2}) \Theta(-E_{k_2}) \Theta(E_{p_1}) \Theta(E_{p_2}) \tag{S32}$$

(for the purpose of the present estimate we take $T = 0$; the role of finite temperature is played instead by $E_{k_1} > 0$). The step functions here indicate that a collision happened with an electron below the Fermi level, $E_{k_2} < 0$, and that $E_{p_{1,2}} > 0$ in the final state. Parameter $M^{k_1,k_2}_{p_1,p_2}$ is the collision matrix element which we now compute. We focus on the long wavelength limit and thus assume a contact repulsion between electrons, $H_{\rm int} = V_{\rm ee} \int dx\, \eta^\dagger_\downarrow(x) \eta_\downarrow(x) \eta^\dagger_\uparrow(x) \eta_\uparrow(x)$. In addition, we account for a static disorder potential $U(x)$ — as discussed in the main text, pair collisions would be forbidden were the edge translationally invariant. By computing the T-matrix [S2] perturbatively in $V_{\rm ee}$ and $U(x)$, we find for the matrix element to the lowest non-vanishing order:

$$\begin{aligned} M^{k_1,k_2}_{p_1,p_2} &= \frac{1}{\mathcal{L}^2} V_{\rm ee} U_{p_1+p_2-k_1-k_2} \Big[\frac{1}{E_{p_1} - E_{k_1+k_2-p_2}} + \frac{1}{E_{p_2} - E_{k_1+k_2-p_1}} + \frac{1}{E_{k_1} - E_{p_1+p_2-k_2}} + \frac{1}{E_{k_2} - E_{p_1+p_2-k_1}} \Big] \\ &\approx \frac{1}{\mathcal{L}^2} \frac{V_{\rm ee} U_{p_1+p_2-k_1-k_2}}{\tilde{m} v^2} \sim \frac{1}{\mathcal{L}^2} \frac{e^2}{\kappa} \frac{U_{p_1+p_2-k_1-k_2}}{\hbar\omega_{\rm c}} \end{aligned} \tag{S33}$$

Here $\mathcal{L}$ is the normalization length and U_k is the Fourier transform of $U(x)$. We estimated $\tilde{m} \sim \frac{\hbar\omega_{\rm c}}{v^2}$ and $V_{\rm ee} \sim \frac{e^2}{\kappa}$, where κ is the dielectric constant of the environment. Substituting expression (S33) in Eq. (S32), evaluating sums over momenta, and averaging the resulting rate over disorder, we obtain:

$$\tau_{\rm in}^{-1}(E_{k_1}) = b\, E_{k_1}^2, \qquad b \sim \left[\frac{e^2}{\kappa\hbar v}\right]^2 \frac{1}{(\hbar\omega_{\rm c})^2} \frac{1}{\hbar^2 v} \int dx' \langle U(x) U(x') \rangle. \tag{S34}$$

We see that the coefficient b depends very sensitively on disorder. If the latter only comes from the electron excursions into the dirty superconductor, then $\langle U(x)U(x')\rangle = (\hbar v)^2 \delta(x - x')/l_{\rm A}$ and

$$b \sim \left[\frac{e^2}{\kappa\hbar v}\right]^2 \frac{1}{(\hbar\omega_{\rm c})^2} \frac{v}{l_{\rm A}}. \tag{S35}$$

In this case, we can estimate $T_{\rm in} \sim \hbar\omega_{\rm c}[\kappa\hbar v/e^2](l_{\rm A}/L)^{1/2}$; its comparison with the presented in the main text estimate for $T_{\rm sm}$ depends strongly on $\Delta/(\hbar\omega_{\rm c})$.

[S1] A. A. Ovchinnikov and N. S. Erikhman, *Temperature and frequency dependence of the electron conductivity in a two-band model with impurities*, Sov. Phys. JETP **51**, 4, 728 (1980).

[S2] J. M. Ziman, *Elements of Advanced Quantum Theory*, Cambridge University Press, Cambridge, England (1969).

[S3] L. Zhao, E. G. Arnault, A. Bondarev, A. Seredinski, T. F. Q. Larson, A. W. Draelos, H. Li, K. Watanabe, T. Taniguchi, F. Amet, H. U. Baranger, G. Finkelstein, *Interference of chiral Andreev edge states*, Nature Physics **16**, 862 (2020).

[S4] V. V. Schmidt, *The Physics of Superconductors: Introduction to Fundamentals and Applications*, Springer-Verlag Berlin (1997).

[S5] T. Ando and U. Yasutada, *Theory of Quantum Transport in a Two-Dimensional Electron System under Magnetic Fields. I. Characteristics of Level Broadening and Transport under Strong Fields*, J. Phys. Soc. Jpn. **36**, 959 (1974).

[S6] B. I. Halperin, *Quantized Hall conductance, current-carrying edge states, and the existence of extended states in a two-dimensional disordered potential*, Phys. Rev. B **25**, 2185 (1982).
